# A New Photoacoustic Soot Spectrophone for Filter-Free Measurements of Black Carbon at 880 nm

**DOI:** 10.3390/molecules27186065

**Published:** 2022-09-16

**Authors:** Goufrane Abichou, Soulemane H. Ngagine, Tong N. Ba, Gaoxuan Wang, Pascal Flament, Karine Deboudt, Sébastien Dusanter, Markus W. Sigrist, Alexandre Tomas, Weidong Chen

**Affiliations:** 1 Laboratoire de Physicochimie de l’Atmosphère, Université du Littoral Côte d’Opale, 189A Avenue Maurice Schumann, 59140 Dunkerque, France; 2 Center for Energy and Environment, IMT Nord Europe, Institut Mines-Télécom, Univ. Lille, 941 Rue Charles Bourseul, 59500 Douai, France; 3 Laser Spectroscopy and Sensing Laboratory, Institute for Quantum Electronics, ETH Zurich, Otto-Stern-Weg 1, 8093 Zurich, Switzerland

**Keywords:** light absorption, photoacoustic spectroscopy, black carbon

## Abstract

A new photoacoustic soot spectrometer (PASS) operating at 880 nm was developed, for the first time, for filter-free measurements of black carbon (BC). The performance of the developed PASS was characterized and evaluated using a reference aethalometer AE51 on incense smoke in the air. An excellent correlation on the measurement of incense smoke was found between the two instruments in comparison with a regression coefficient of 0.99. A 1 σ detection limit of 0.8 µg m^−3^ was achieved for BC measurement at a time resolution of 1 s. It can be further reduced to 0.1 µg m^−3^, using a longer integration time of 1 min.

## 1. Introduction

Black carbon (BC) is one of the main short-lived climate pollutants [1]. It is a relatively pure form of carbon, also known as soot [2]. It is formed during an incomplete combustion of biofuels, fossil fuels and biomass products [1]. It is considered to be the second cause of global warming after CO_2_ with a radiative forcing of 1.1 W m^−2^ against 1.7 W m^−2^ for CO_2_ [3]. Besides its impact on the climate, BC also has detrimental effects on human health, causing respiratory and cardiovascular diseases, as well as lung cancers and premature mortalities [1]. BC also has an indirect impact on agriculture. For instance, it modifies rainfall patterns and disturbs monsoons in many parts of Asia and Africa, which are critical for crop yields [4]. In addition, when it comes into direct contact with vegetation, it reduces sunlight by surface deposition on leaves, and consequently decreases the net photosynthetic rate [5].

Since BC has a short atmospheric lifetime (days to weeks) [2], it is possible to rapidly intervene to decrease its emission, thus, reducing its impact on ecosystems, human health and climate [6]. Among the techniques used for measuring BC, there are mainly three methods: (1) thermal optical analysis, measuring BC mass concentration with the advantage of distinguishing organic and elemental carbon [7]; (2) laser-induced incandescence measuring both particle size and mass concentrations [8] and (3) aethalometers based on direct absorption spectroscopy, commercially available for filter-based measurements of light absorption by BC to determine its concentrations [9]. However, these techniques present significant uncertainties that can reach 20–30%, due to sampling artifacts and biases [10].

Recently, photoacoustic absorption spectroscopy (PAS) was shown to be a valuable technique for direct filter-free measurements of BC, with advantages of high accuracy and portability, and a lower sensitivity to light scattering losses as in filter-based methods [11]. The first photoacoustic soot spectrometer (PASS) dedicated to the detection of BC was presented by Petzold and Niessner in 1996 [12]. The authors used a diode laser which was operated at 802 nm with an optical power of 450 mW. Later on, various PAS instruments operating in the UV–vis and near-IR regions were developed for experimental studies of soot [6,11], as well as for industrial applications [13]. This technique was even deployed on aircraft platforms [14,15], and was commercialized as a multiple wavelength photoacoustic spectrophones: PASS-3 (Droplet Measurement Technologies, Boulder, CO, USA) [16].

In this publication, we report on the development of a PASS operating at 880 nm for the measurement of BC concentrations with a fast time resolution of 1 s. The performances of this instrument were characterized and evaluated using a reference aethalometer and samples consisting of incense smoke in the air.

It is worth noting that the present instrument is the first PA spectrophone developed for the measurement of BC at 880 nm, after the one reported in 2007 that operated at 870 nm for the measurement of BC with a resolution time of 2 min (LoD not reported) [17]. The advantage of choosing this wavelength is that BC is detected without interferences from other types of absorbing particulate species, especially organic carbon [18]. Indeed, 880 nm is the same wavelength used for the reference aethalometer AE51, while the reference instrument uses the filter-based optical measurement method, which is the main limiting factor to the current instrumental measurement accuracy.

This work demonstrates that photoacoustic spectroscopy is a valuable technique, offering the unique capacity of direct and filter-free measurement of absolute light absorption by black carbon, with advantages of high accuracy, high sensitivity, and high portability.

## 2. Results

### 2.1. Materials and Methods

#### 2.1.1. Measurement of Particle Absorption by Photoacoustic Spectroscopy

PAS is an analytical technique suitable for absolute absorption measurements of trace gases and particles [6], with advantages of a simple setup and relatively low cost. This technique relies on the measurement of an acoustic signal resulting from the absorption of photons of a modulated light by the targeted species that absorbs photons at specific wavelengths. The recorded signal can then be used to provide information on the absorption properties of the detected species (absorption coefficients and concentration) [6].

The photoacoustic (PA) effect resulting from the absorption of photons by gaseous species or aerosol particles results from [19] (i) localized heat release as a result of the absorbed energy; (ii) periodical thermal expansion of the sample due to the modulation of the exciting light; (iii) the generation of an acoustic signal whose intensity is proportional to the absorbed energy.

The acoustic signal is then quantified using suitable acoustic transducers such as microphones. The measured PA signal S (V) can be expressed using the following equation [6]:

S = P × M × C_cell_ × α_0_ × C + S_b_ (1)

where P (W) is the incident light power, M (V/Pa) the microphone sensitivity. C_cell_ (Pa m W^−1^) is the PA absorption cell constant expressing the conversion efficiency of the optical energy into an acoustic energy [13], the latter being a quality indicator for the PA cell [20]. C_cell_ is independent of the measured absorber (gas or particles) [11]. α_0_ (Mm^−1^ ppb^−1^, Mm^−1^ = 10^−6^ m^−1^) is the specific absorption coefficient of the detected trace gas and C (ppbv) is its concentration. In the case of particle measurements, α_0_ (m^2^ g^−1^) is the aerosol absorption mass coefficient and C (μg m^−3^) is the aerosol mass concentration [6]. S_b_ (V) is the background PA noise arising from ambient acoustic noise and is generated by photons hitting the walls/windows of the PA cell.

#### 2.1.2. Experimental Setup

**Figure 1:**
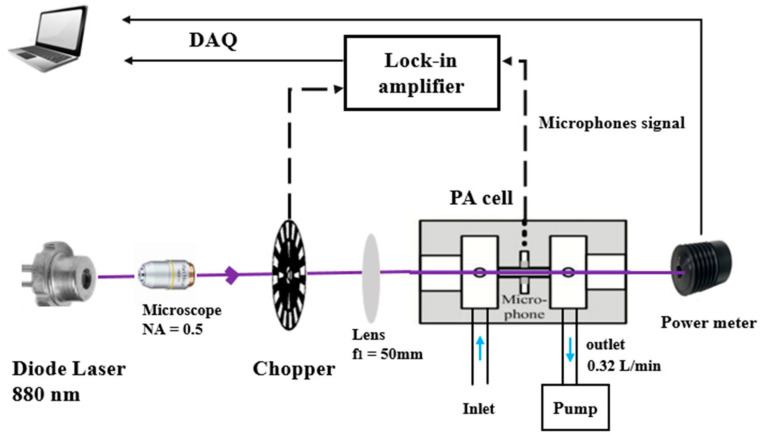
Schematic of the PASS instrument for BC measurements.

The PASS is composed of four main parts: (1) a modulated light source emitting at a specific wavelength, (2) a sampling cell equipped with an acoustic resonator to enhance the PA signal, (3) an acoustic transducer device for the detection of the acoustic signal consisting of microphones, and (4) an electronic unit for signal amplification and data processing [21]. A schematic of the instrument is shown in Figure 1. It includes a laser source, a PA resonator (PA cell incorporating 2 buffer volumes to reduce the background noise resulting from (i) the external environment, (ii) the flowing gas, and (iii) light absorption on the cell windows) and a data processing module.

The light source is a high power TO-3 laser diode (WaveSpectrum, AL0880F1000, Beijing, China), emitting one line in the spectral range of 880 ± 5 nm with an emission linewidth of ~1 nm. Its maximum output optical power is 1 W powered with a current of 1300 mA and a voltage of 2.2 V. These parameters are controlled by a diode laser controller (6340 ComboSource, Arroyo Instruments, San Luis Obispo, CA, USA). A microscope with a numerical aperture (NA) of 0.5 is used to reshape the laser beam and to focus it on a 100-slot mechanical chopper (New focus 3501, Newport/New Focus, Irvine, CA, USA). The chopper modulates the laser light at the resonance frequency of the acoustic resonator. The modulated laser beam is collimated using a lens with a focal length of 50 mm, and then focused into the acoustic resonator in the PA cell. The laser beam at the output of the cell is collected by a power meter (Coherent, Field Master GS, Saxonburg, PA, USA) to monitor the laser power. Four electret microphones (EK-23329-P07, Knowles, Itasca, IL, USA) are set up in the middle of the acoustic resonator to detect the PA signal. These microphones exhibit a sensitivity of 22.4 mV/Pa at sound frequencies ranging from 100 to 10,000 Hz.

The PA signal is first demodulated at the modulation frequency of the laser light using a lock-in amplifier (SR 830, Stanford Research Systems, Sunnyvale, CA, USA). The signal is then sampled using a data acquisition card (National Instrument PCI-6251, Austin, TX, USA) and a laptop allowing real-time data processing and display via a Labview program. The quantified acoustic signal is finaly normalized by the measured laser power.

To characterize and optimize the performances of the PASS instrument, a series of experiments were performed to optimize the operating parameters, including the modulation frequency and the sampling flow rate, and to evaluate the effect of ambient humidity on the PA signal.

#### 2.1.3. Calibration of the PA Cell

To calibrate the photoacoustic cell, an absorber (gas or particulate matter) with a known concentration and absorption cross section was needed [22]. In the visible up to the near-infrared regions, NO_2_ is commonly used for this purpose [23,24,25]. At 880 nm, NO_2_ exhibits a small absorption, therefore, it is essential to work at a relatively high concentration. Overnight flushing of the PA cell with N_2_ at the end of the experiment was performed to avoid its contamination.

Equation (1) was rearranged to derive C_cell_ from calibration experiments. The measured PA signal (S) for a NO_2_ mixing ratio of 3000 ± 60.6 ppm and the background signal (S_b_) observed when only pure N_2_ was passed through the cell are 25.34 µV (±0.20 µV) and 16.28 μV (±0.22 µV), respectively. The specific absorption coefficient α_0_ (Mm^−1^ ppm^−1^) of NO_2_ was derived from the following equation:

(2) $$\alpha_{0}=\frac{N\;\times \;\sigma \left(\lambda \right)}{C_{{\mathrm{NO}}_{2}}\;}$$

where N = 7.5 × 10^16^ molecules cm^−3^ is the NO_2_ number concentrations at T = 293.5 K and P = 1 atm, and C_NO2_ = 3000 ± 60.6 ppm is the NO_2_ mixing ratio. According to the MPI-Mainz UV/VIS Spectral Atlas of Gaseous Molecules of Atmospheric Interest, the absorption cross section σ(λ) of NO_2_ at 880 nm is 3.51 × 10^−23^ cm^2^ molecules^−1^ [26]. The specific absorption coefficient α_0_ of NO_2_ was then found to be 0.088 Mm^−1^ ppm^−1^. The PA cell constant was deduced to be C_cell_ = 2.21 ± 0.05 Pa m W^−1^. The uncertainty for the cell constant was calculated from a quadratic propagation of errors associated to the PA signal (precision), the NO_2_ mixing ratio, and the laser power. Where $\frac{{\Delta S}_{\mathrm{PA}}}{S_{\mathrm{PA}}}$ = 0.8% (from the lock-in amplifier) is the relative uncertainty of the PA signal, $\frac{{\Delta C}_{{\mathrm{NO}}_{2}}}{C_{{\mathrm{NO}}_{2}}}$ = 2% is the relative uncertainty in the NO_2_ concentration, and $\frac{\Delta P}{P}$ = 0.10% is the uncertainty in the laser power ΔP. The uncertainties from the microphone sensitivity M and the specific absorption coefficient α_0_ are considered negligible. A value of 2.2% was estimated as the relative uncertainty of the cell constant, corresponding to a ${\Delta C}_{\mathrm{cell}}$ = 0.05 Pa m W^−1^.

#### 2.1.4. Modulation Frequency Optimization

The resonant PAS approach is widely employed to enhance the signal-to-noise ratio (SNR) of the PA signal for which an acoustic resonator is used. The frequency of the laser beam modulation should be well matched to the resonance frequency of the acoustic resonator. The resonance frequency of the acoustic resonator can be expressed by the following equations:

(3) $$f=\frac{c}{2\times \left(L\;+\;\Delta L\right)}$$

(4) $$\Delta L=\frac{16}{3\Pi }\;\times \;R$$

where c (m s^−1^) is the sound speed, L (m) is the length of the resonator, ΔL is the end correction factor, and R (m) is the resonator radius [21].

For an ideal gas, the sound speed c can be expressed as follows:

(5) $$c=\sqrt{\frac{Ɣ\times \;p}{\rho }}$$

where Ɣ is the adiabatic index, also known as the isentropic expansion factor, and p (Pa) and ρ (kg m^−3^) are the gas pressure and density, respectively. The sound speed c is, therefore, dependent on the medium composition; in particular, on the nature of the most abundant species present in the sampled gas, i.e., N_2_, O_2_, and to some extent H_2_O in ambient air.

To experimentally determine the resonance frequency of the PASS instrument, PA signals were monitored vs. modulation frequencies for three types of carrier gases:

1. Nitrogen (N_2_) with low water content (RH = 9.5%, T = 27 °C)—Referred to as dry N_2_ in the following sections;
2. Compressed air with low water content (RH = 12%, T = 27 °C)—Referred to as dry compressed air in the following sections;
3. Humid filtered indoor ambient air (RH = 34%, T = 27 °C), filtered by a PTFE membrane filter (0.2 μm) to remove particles, assuming that the mass concentration of particles with a size less than 0.2 μm is negligible (<1 μg.m^−3^) given their small size; hence, their effects on the PA signal are also negligible.

It is worth noting that a photoacoustic signal is observed during these experiments, even in the absence of BC, due to the absorption of photons by water vapor, as discussed in Section 2.1.5.

**Figure 2:**
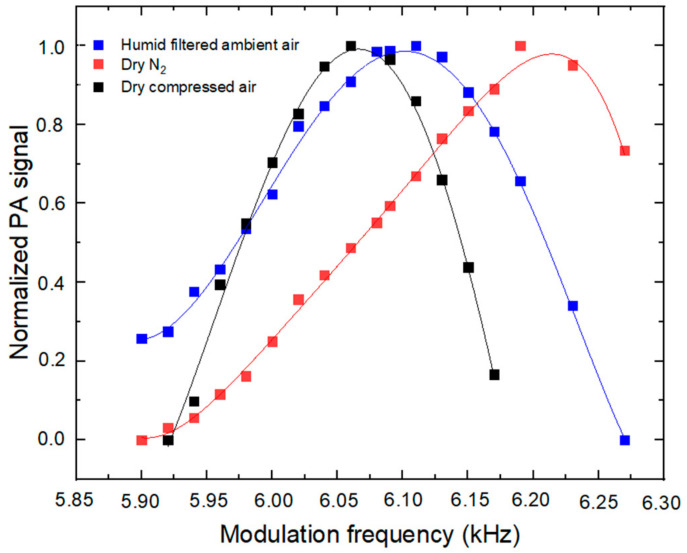
Dependence of the PA signal on the modulation frequency.

Figure 2 shows the normalized PA signals at modulation frequencies ranging from 5.90 to 6.27 kHz. Based on Equations (4) and (6), the calculated resonance frequencies are 6.21 kHz for dry N_2_ and 6.05 kHz for dry compressed air, given a resonator length of 23 mm and a radius R of 3 mm, and a sound speed of 349 and 340 m s^−1^ in N_2_ and air, respectively (Ɣ = 1.4, p = 1.013 × 10^5^ Pa, ρ (N_2_) = 1.15 kg m^3^, ρ (air) = 1.21 kg m^3^). Consistently, the experimental resonance frequencies (6.19 kHz for N_2_ and 6.06 kHz for dry air) are within 0.3% of the theoretical values. A modulation frequency of 6.06 kHz (for measurements in air) was chosen as the operating parameter for further experiments and field applications.

It is clear from Figure 2 that water vapor has an impact on the resonance frequency [27] and should be taken into consideration for ambient measurements. In fact, at 6.06 kHz, an increase in the RH from 12 to 34% would lead to a relative decrease in the instrument response of approximately 10%, which is significant.

#### 2.1.5. Impact of Relative Humidity on PA Signal

The PA signal depends on the vibration-to-translation (V-T) relaxation rate of the target absorber [27]. In this regard, water vapor is considered as a promoter to accelerate this process and it can considerably enhance the PA signal [28]. In addition, if H_2_O vapor absorbs the laser light at the operating wavelength, it will further “increase” the PA signal due to its direct absorption. According to the HITRAN database, H_2_O vapor presents an absorption line at 880 nm with a cross section of ~10^−27^ cm^2^ mol^−1^. Considering the high H_2_O concentrations in the atmosphere, H_2_O absorption may lead to a significant interference on the measurements of PA signals.

**Figure 3:**
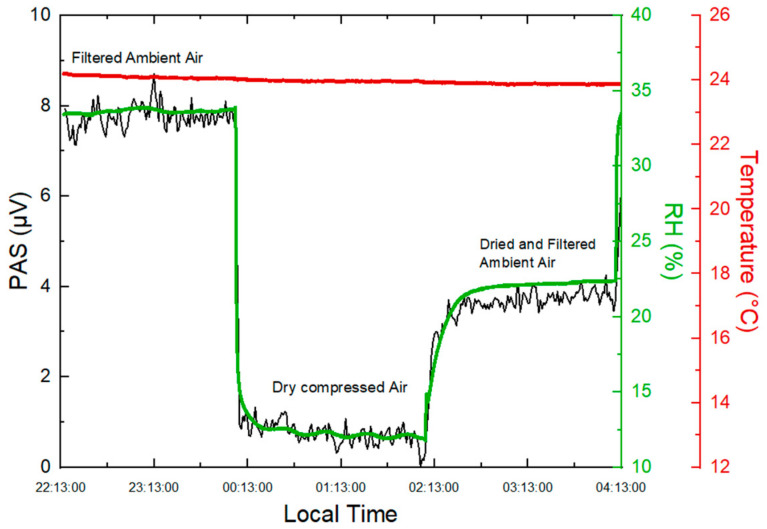
Time series of PAS measurements from filtered ambient air, dry compressed air, and dried and filtered ambient air.

In the present work, the impact of H_2_O vapor was experimentally investigated. Ambient RH was monitored using a temperature and humidity sensor (Sensirion, SHT71, Stäfa, Switzerland) connected to the outlet of the PA cell. Increasing RH compressed air by 22% at a constant temperature results in an increase of 7 µV in the PA signal (Figure 3), which would be equivalent to a concentration of 32.13 µg m^−3^ of BC on the basis of the sensitivity reported in Section 2.2.

In order to reduce the impact of ambient humidity, a Nafion dryer (Perma pure, 30 cm monotube MD-110-12S-4 dryer, Lakewood, NJ, USA) was used to dry the samples before the inlet of the PA cell. Dry compressed air was used as purge gas at an optimal flow rate of 4 L min^−1^, as recommended by the manufacturer.

An overnight experiment was carried out to monitor the variation of the PA signal depending on the change in RH. The sampling was controlled automatically with a Labview-based program to measure the PA signals resulting from three different carrier gases: dry compressed air, ambient air filtered with a PTFE membrane filter, and ambient air dried by the Nafion dryer (also particle-filtered with a PTFE filter). A reduction in RH from 34% to 22% was observed after drying ambient air by the Nafion dryer, as shown in Figure 3.

In the present work, the use of the Nafion dryer allowed to stabilize RH at a constant level of 22% in the samples and the PA signal generated by residual RH was considered as a background signal that was then subtracted from BC measurements in ambient air to deduce the absorption generated only from BC (effective PA signal: S_EPA_).

#### 2.1.6. Sampling Flow Rate

**Figure 4:**
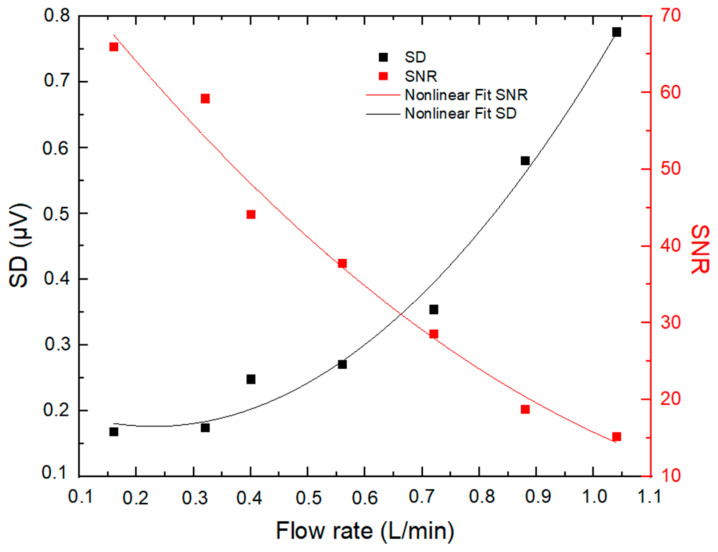
Dependence of the PA noise (Standard Deviation—SD of 1 s blank measurements) and SNR of the PA signal on the sampling flow rate.

For PA measurements regarding the majority of other measurement techniques, the noise level is the limiting factor to the quantification of low concentrations [19]. Several factors can contribute to the noise in PA measurements, one major contribution coming from acoustic noise generated from turbulence when air is sampled through the PA cell. The dependence of the noise level in PA signal (standard deviation, SD, observed during blank measurements) and the SNR of the PA signal on the sampling flow rate were investigated within the range of approximately 0.1–1 L min^−1^ using particle-filtered ambient air (RH = 33 ± 1%, T = 28 °C), as shown in Figure 4.

It has been noticed that noise increases with the flow rate in a nonlinear way, while the SNR exhibits inverse behavior, decreasing with higher flow rates. In this regard, it is essential to indicate that, in the PA system, above a certain value of the flow rate (typically about 0.5 L min^−1^ [19] and 0.4 L min^−1^ in our case), the flow becomes turbulent and generates large acoustic noise that degrades the SNR of the PA signal [20]. According to our investigation, a sampling flow rate of 0.32 L min^−1^ was selected as a good tradeoff between SNR and air residence time in the PA resonator (0.12 s).

### 2.2. Evaluation of the PASS Performances

Side-by-side measurements of BC emitted from incense smoke have been performed using the PASS instrument and a reference aethalometer (microAeth, AE51). This experiment allowed for the following: (1) calibration of the PASS instrument; (2) checking of the linearity of the PA signal with BC concentration; (3) estimation of the limit of detection (LoD) of the PASS for BC measurements in ambient air.

**Figure 5:**
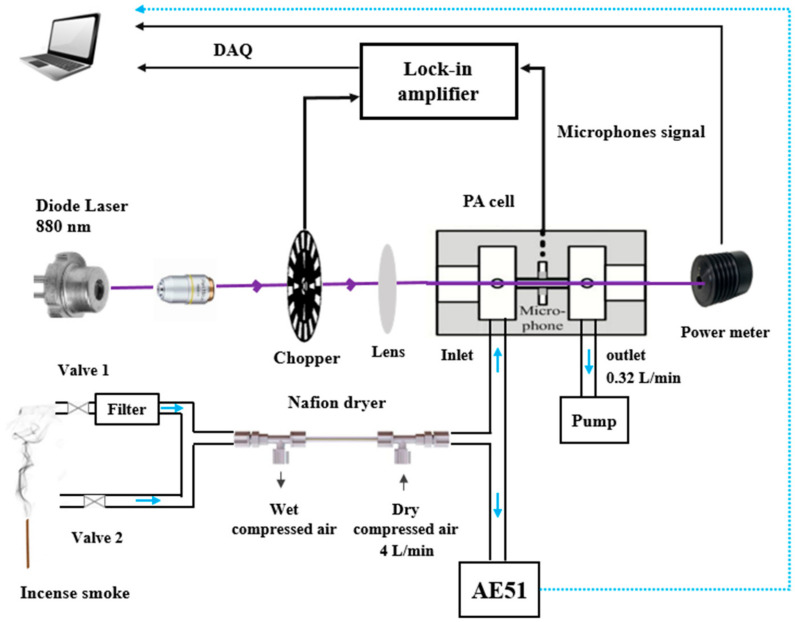
Schematic of the experimental setup for the measurement of incense generated BC with the PASS instrument and the reference aethalometer (AE51).

Burning incense in indoor ambient air led to the formation of BC with concentrations ranging from 0 to 200 µg m^−3^. The reference aethalometer is a filter-based spectrometer operating at 880 nm. At 880 nm, the absorption is interpreted as BC deposition on the filter [18]. Its operation principle is based on optical measurement of light absorption by the particles collected on a filter. The BC concentration can be measured in the range of 0–1 mg m^−3^. A schematic presentation of the experimental setup is shown in Figure 5. The inlets of both PASS and aethalometer were connected to a Nafion dryer, where dry compressed air was injected in the countercurrent at a flow rate of 4 L min^−1^ in order to reduce RH and maintain its stability at approximately 20%. As mentioned above, this setup is required to avoid the impact of humidity changes on the PA signal.

**Figure 6:**
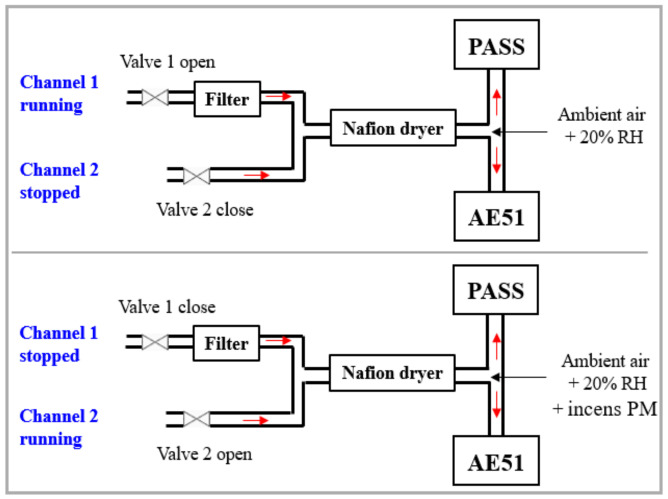
Schematic representation of the 2-channel measurement approach.

**Figure 7:**
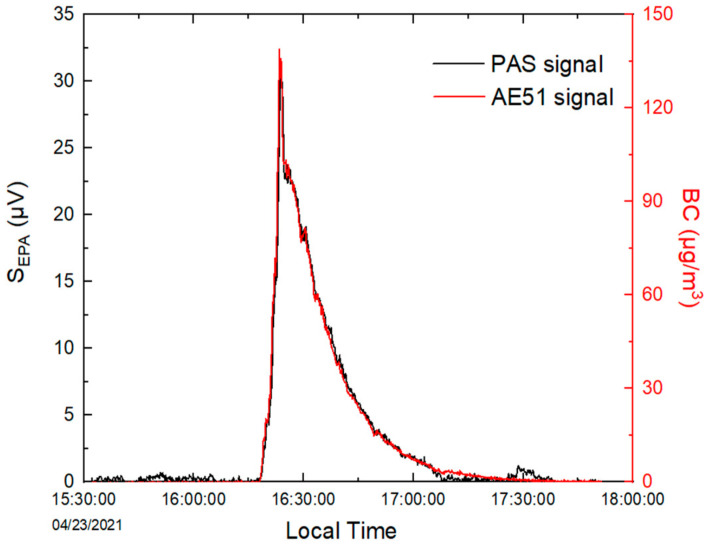
Time series of measurements for incense-generated BC by PASS and AE51.

To obtain the PA signal resulting only from BC absorption without the contribution of gas species in air, a two-channel measurement method (presented in Figure 6) was carried out: (1) channel one, equipped with a particle matter (PM) filter, allows for the measuring of the potential contribution of gas species in air and was considered for background measurements; (2) channel two, without a PM filter, allows for the measuring of the contribution of both BC and gas species in air. The difference in signal between the two channels represents the absorption of BC (S_EPA_ in Figure 7).

**Figure 8:**
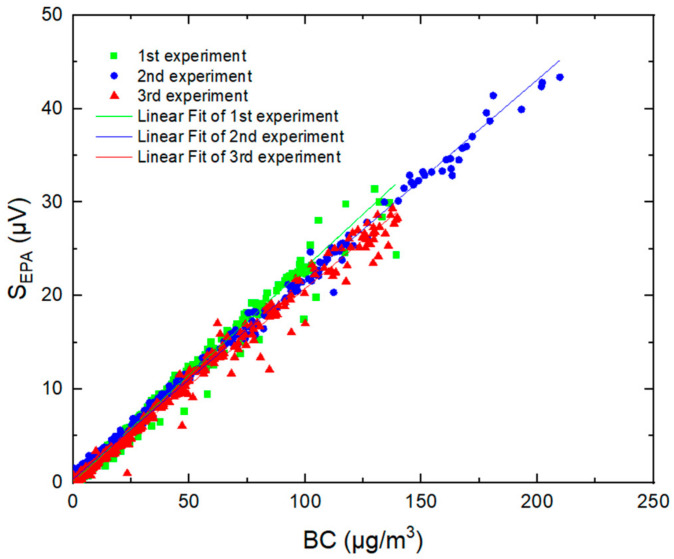
PA signals from the PASS versus BC mass concentrations measured by the AE51 during three experiences.

Time series of PASS measurements for incense-generated BC at a time resolution of 1 s (averaged to 10 s) are shown in Figure 7, together with 10 s measurements from the aethalometer. The PA signal was found to be well correlated with BC concentrations measured by the AE51, with a linear regression coefficient of 0.99 in the range of LoD up to 200 μg m^−3^ (green plot Figure 8). The repeatability of the measurements was also evaluated by reproducing the same experiment two more times. Figure 8 shows good repeatability, with slopes ranging from 0.207 to 0.229 μV/(μg m^−3^).

The LoD was estimated using the following equation:

LoD = SD/S_Slope_ (6)

where S_slope_ (μV/(μg m^−3^)) is the slope of the linear regression between the PA signal and the BC concentration, also called sensitivity (shown in Figure 8). Using SD = 0.20 μV determined from blank measurements (RH = 21%, T = 24 °C) and slopes derived from the calibration experiments, the 1 σ minimum detectable mass concentration (SNR = 1) was evaluated to be in the range of 0.75–0.86 µg m^−3^ at a time resolution of 1 s (that can be enhanced to 0.1 μg.m^−3^ when working at a time resolution of 1 min). This limit of detection shows that the PASS instrument is suitable for measurements of BC in the troposphere, especially in areas where BC concentrations vary from 12 μg m^−3^ [29] to 60 μg m^−3^ [30].

Wavelength-dependent mass absorption coefficient (α_MAC_) of the incense particles can be estimated using the following equation:

(7) $$\alpha_{\mathrm{MAC}}=\frac{S_{\mathrm{slope}}}{P\;\times \;M\;\times {\;C}_{\mathrm{cell}}}$$

where S_slope_ = 0.217 µV/(µg m^−3^) (derived from the average of the three fits in Figure 8) and P = 174 mW. The value of α_MAC_ was determined to be 6.3 ± 0.44 m^2^ g^−1^, which corresponds to a minimum measurable absorption coefficient of 5.23 Mm^−1^ ± 0.36 (1 σ) (=LoD × α_MAC_).

The uncertainty associated with the mass absorption coefficient was derived from a quadratic propagation of errors from the precision of the PA signal, the power measurement accuracy, and errors associated to the cell constant and the fluctuations in BC concentrations. The latter was calculated from the aethalometer measurements using the following equation [31]:

(8) $$\frac{\Delta \mathrm{BC}}{\mathrm{BC}}=\sqrt{\begin{array}{c} {\left(\frac{{\Delta \sigma }_{\mathrm{ATN}}}{\sigma_{\mathrm{ATN}}}\right)}^{2}+{\left(\frac{\Delta A}{A}\right)}^{2}+{\left(\frac{\Delta Q}{Q}\right)}^{2}+{\left(\frac{\Delta \mathrm{dt}}{\mathrm{dt}}\right)}^{2}\\ +2{\left((\frac{{\Delta I}_{0}}{I_{0}})\right)}^{2}+2(\left(\frac{\Delta I}{I}{)}^{2}\right) \end{array}}$$

where $\frac{{\Delta \sigma }_{\mathrm{ATN}}}{\sigma_{\mathrm{ATN}}}$ is the relative uncertainty from the attenuation cross section; it is assumed to be negligible for the microAeth^®^ AE51 [31]. $\frac{\Delta A}{A}$ = 2% is the uncertainty from the spot area on the filter [31]. $\frac{\Delta Q}{Q}$ = 5% is the uncertainty related to the sampling flow rate [31]. $\frac{\Delta \mathrm{dt}}{\mathrm{dt}}$ = 0% is the measurement time uncertainty [31]. $\frac{{\Delta I}_{0}}{I_{0}}$ = 2.1% and $\frac{\Delta I}{I}$ = 1.55% are, respectively, the uncertainties of the reference signal (= SD/mean of the reference signal) and the sensing signal (= SD/mean of the sensing signal of the entire measurement). The relative uncertainty from the fluctuation in BC concentration was evaluated to be 6.5%. The relative uncertainty in α_MAC_ was found to be 6.9%, which corresponds to Δα_MAC_ = 0.44 m^2^ g^−1^.

The obtained values of the mass absorption coefficient and the minimum measurable absorption coefficient are in good agreement with those reported by [32] consisting of 7.5 ± 1.2 m^2^ g^−1^ and 6.23 ± 1 Mm^−1^, respectively.

However, the obtained results are higher than those calculated on the basis of a power law, as shown in the following equation [33]:

(9) $$\alpha_{\mathrm{MAC}}\;=\;k_{0}\;\times \;(\frac{\lambda }{500\;\mathrm{nm}}{)}^{-\mathrm{AAE}}$$

where k_0_ (=7 ± 0.4 m^2^ g^−1^) is a constant including the aerosol mass concentration and AAE (=1.2 ± 0.4) is the Absorption Angstrom Exponent (an important parameter to characterize the variation of the aerosol absorption with respect to the wavelength). α_MAC_ was found to be 3.55 ± 1.17 m^2^ g^−1^, corresponding to a minimum measurable absorption coefficient of 2.94 ± 0.97 Mm^−1^. The difference between the experimental values and the theoretical ones is likely due to a low fraction in the soot considered by the theoretical equation (valid for aerosols in general) [33].

## 3. Conclusions and Outlooks

A photoacoustic soot spectrophone operating at 880 nm was developed for the measurement of black carbon. This instrument has the advantage of low uncertainties compared to the filter-based techniques routinely used for aerosol measurements, and has a faster time resolution (1 s) compared to aethalometers (usually operating at 10 s).

The calibration of this instrument was performed by analyzing BC emitted from incense smoke using an aethalometer as a reference instrument. The sensitivity factor derived from these calibration experiments is 0.22 μV/(μg m^−3^). Taking into account the measurement noise, which was minimized through an optimization of the sampling flow rate and a reduction of RH in the sample, it has led to a 1 σ LoD (SNR = 1) of approximately 0.8 µg m^−3^ at a time resolution of 1 s. This LoD can be improved to 0.1 μg m^−3^ using a longer integration time of 1 min.

Further improvements in the sensitivity of the current PASS instrument can be achieved by increasing the number of microphones in the PA cell and using higher laser power. The RH effects can be further reduced using a longer and more efficient Nafion dryer.
